# P-1800. Saliva as a Promising Additional Specimen for Enhanced RSV Detection in Pediatric Patients; A New Vaccine Surveillance Network Study

**DOI:** 10.1093/ofid/ofaf695.1969

**Published:** 2026-01-11

**Authors:** Anjana Sasidharan, Montserrat Santos, Varun chandra Boinpelly, Dithi Banerjee, Brian R Lee, Jennifer E Schuster, Dinah Dosdos, Mary E Moffatt, Kirsten L Weltmer, Gina Weddle, Casey M Kalman, Heidi L Moline, Rangaraj Selvarangan

**Affiliations:** Childrens Mercy Hospital, Missouri, Kansas; Children's Mercy Research Hospital, Kansas City, Missouri; CMRI, Overland Park, Kansas; Children's Mercy Hospital, Overland Park, KS; Children's Mercy Kansas City, Kansas City, Missouri; Children's Mercy Kansas City, Kansas City, Missouri; Children's Mercy Hospital Kansas City, Kansas City, Missouri; Children's Mercy Kansas City, University of Missouri Kansas City School of Medicine, Kansas City, Missouri; Children's Mercy Kansas City, Kansas City, Missouri; Children's Mercy Hospital, Overland Park, KS; Centers for Disease Control and Prevention, Washington, District of Columbia; US-CDC, Atlanta, Georgia; Children’s Mercy Hospital, Kansas City, Missouri

## Abstract

**Background:**

Respiratory syncytial virus (RSV) detection is essential for pediatric acute clinical care. While nasopharyngeal (NPS) and mid-turbinate swabs (MTS) remain the gold standard, they may be distressing to children. Saliva has shown promise as a diagnostic specimen in adults for RSV, but its diagnostic utility in children is unknown. In this study, we evaluated the performance of saliva compared to paired MTS samples for RSV detection in children.

**Table 1.**
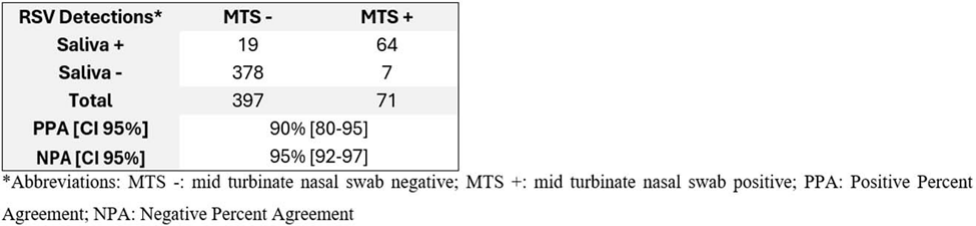
Detections of respiratory syncytial virus A/B in paired mid-turbinate swabs and saliva specimens, December 2024-March 2025

**Table 2.**
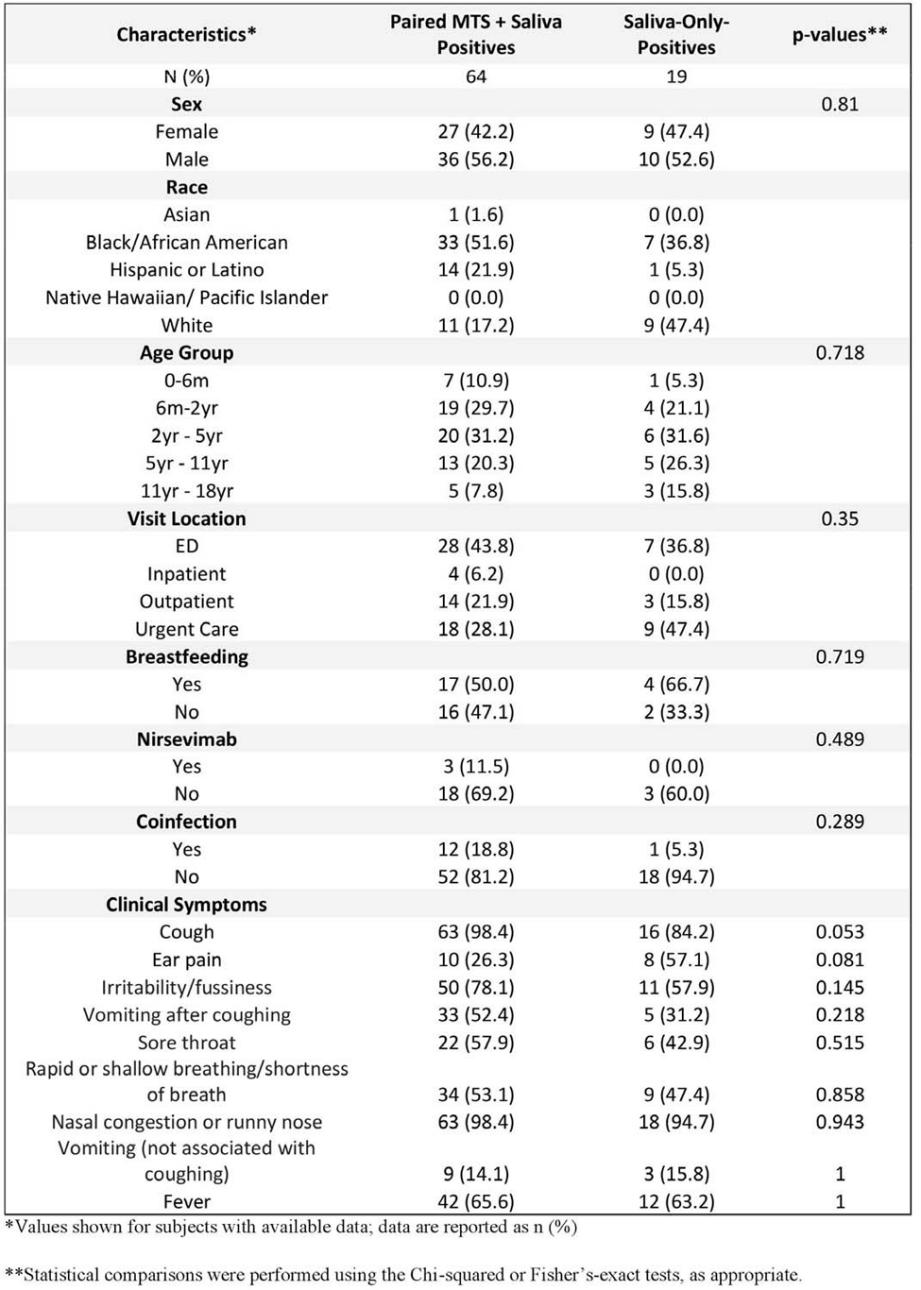
Patient demographics and clinical characteristics of paired mid-turbinate swab and saliva RSV positives versus saliva-only positive cases

**Methods:**

Subjects aged < 18 years with acute respiratory symptoms were enrolled from December 2024 to March 2025 by the New Vaccine Surveillance Network. Saliva was obtained using the SalivaBio Children’s swab on the same day as MTS collection. MTS was tested using QIAstat-Dx Respiratory SARS-COV2 panel and saliva was extracted via Kingfisher Apex and tested by RSV-single-plex Real-time Polymerase Chain Reaction (RT-PCR). Demographic and clinical data were analyzed, and statistical comparisons were performed using Chi-squared or Fisher’s exact tests, as appropriate.

**Results:**

A total of 786 paired MTS and saliva samples were collected. Among the 468 (59.5%) paired MTS and saliva specimens with sufficient volume, 154 (33%) were positive for RSV in MTS or saliva: 71 (15%) MTS specimens, 83 (18%) saliva specimens. Median (IQR) Ct values were 25.6 (21.6 – 30.9) in MTS and 29.1 (26.0 – 33.1) in saliva. Saliva missed 7 (1.5%) cases detected by MTS (median MTS Ct: 31.3), while detecting 19 (4.1%) additional cases (median saliva Ct: 35.2) suggesting viral persistence in oral secretions (Table 1.). Of these, 13 cases were RSV-B and 6 were RSV-A. Saliva increased diagnostic yield by 20% compared to MTS. Positive and negative percent agreement between both sample types were 90% and 95%, respectively.

Demographic and clinical symptoms were broadly comparable, although cough was less common in the saliva-only group (84.2% vs 98.4%, *p*=0.053). Co-infection was lower in saliva-only group (5.3% vs 18.8%, *p*=0.289), though not statistically different (Table 2).

**Conclusion:**

Saliva showed greater positive percent agreement for RSV detection compared to MTS samples, particularly in cases with higher Ct values suggestive of lower viral loads. These findings support saliva as a complementary specimen, reinforced by the observed 20% increase in diagnostic yield.

**Disclosures:**

Brian R. Lee, PhD, MPH, Merck: Grant/Research Support Rangaraj Selvarangan, PhD, Altona: Grant/Research Support|Biomerieux: Advisor/Consultant|Biomerieux: Grant/Research Support|Biomerieux: Honoraria|Cepheid: Grant/Research Support|Hologic: Grant/Research Support|Hologic: Honoraria|Meridian: Grant/Research Support|Qiagen: Grant/Research Support

